# Androgens and Adipose Tissue in Males: A Complex and Reciprocal Interplay

**DOI:** 10.1155/2012/789653

**Published:** 2011-12-22

**Authors:** Caterina Mammi, Matilde Calanchini, Antonella Antelmi, Francesca Cinti, Giuseppe M. C. Rosano, Andrea Lenzi, Massimiliano Caprio, Andrea Fabbri

**Affiliations:** ^1^Centre of Clinical and Basic Research, Department of Medical Sciences, IRCCS San Raffaele Pisana, 235 00163 Rome, Italy; ^2^Unit of Endocrinology, S. Eugenio & CTO A. Alesini Hospitals, Department of Internal Medicine, University of Rome “Tor Vergata”, Rome, Italy; ^3^Department of Medical Pathophysiology, University of Rome “La Sapienza”, Rome, Italy

## Abstract

Clinical evidence shows that in males obesity is frequently associated with hypogonadism and vice versa; also, low testosterone levels have been considered a “hallmark” of metabolic syndrome in men. These observations indicate that there is a strict connection between anatomically and functionally distinct cell types such as white adipocytes and Leydig cells, that synthesize testosterone. Adipose tissue is able to control several functions of the testis through its products secreted in the bloodstream. On the other hand, circulating levels of testosterone and estradiol deeply affect adipocyte proliferation, differentiation, and fat mass distribution, hereby controlling critical metabolic functions, such as food intake, insulin sensitivity, vascular reactivity, and immunity. This paper highlights the existing clinical and experimental evidence linking androgens and adipose tissue and illustrates the consequences occurring when the balance between fat mass distribution and eugonadism is lost.

## 1. Introduction

Adipose tissue, in addition to its role as a storage for triglycerides, can be considered as an active, atypical endocrine organ [1], given its ability to synthesize and secrete into the bloodstream several hormones. Overall, only thirty percent of adipose tissue is represented by mature adipocytes, given that multiple cell types are present in its contest. In fact, the remaining tissue is represented by multipotent stem cells, nerve tissue, small blood vessels, fibroblasts, and preadipocytes in various stages of differentiation [2]. Importantly, adipose tissue contributes to regulate several functions such as energy balance, food intake and appetite, immunity, insulin sensitivity, blood pressure and reproduction [3], releasing adipokines that have both local and systemic biological effects. Dysfunctional secretion of adipokines and free fatty acids contributes to the development of an inflammatory state and has a causal role for the development of the insulin-resistant state of obesity [4].

Two types of adipose tissue are present in mammals: white adipose tissue (WAT) and brown adipose tissue (BAT). WAT stores energy as triglycerides. In case of lack of energy, such as fasting, lipolysis in WAT causes the release of fatty acids into the plasma to provide fuel for energy generation. Indeed lipases contained in adipocytes transform triglycerides into fatty acid and glycerol that are transported via the blood to the muscle, liver, and BAT to fatty acids oxidation [5]. In contrast, BAT plays a physiological function in adaptive thermogenesis storing triglycerides in multilocular adipocytes that serve as energy reserves easily accessible to heat production and restore energy expenditure induced by cold exposure or diet [6].

Also adipose tissue is distributed unevenly through the body and is represented by two major compartments which are different for distribution and metabolism: subcutaneous and visceral depots [7]. Intra-abdominal or visceral fat induces an increased risk of cardiovascular and metabolic complications, whereas subcutaneous fat exerts some still undefined protective actions [8]. Visceral and subcutaneous adipose tissues express different adipokines. An important role in the pathogenesis of cardiovascular diseases is played by visceral fat, since it expresses many substances strongly involved in cardiovascular diseases, such as leptin, TNF*α*, IL-6, PAI-1, which have a direct access to the liver via portal vein, with a strong impact on the inflammatory processes [9].

Subcutaneous fat is metabolically less active than visceral fat and produces mostly protective substances such as leptin and adiponectin [10] and is less sensitive to glucocorticoids because it has lower levels of glucocorticoid receptor [11–13], thereby it could exert a protective role on metabolic homeostasis, hence counteracting the dysfunctional adipose tissue in visceral and ectopic compartments [14].

Aim of this paper is to review the most recent clinical and experimental evidence linking androgens and adipose tissue and to discuss the reciprocal interplay between obesity and hypogonadism.

## 2. Leptin and Testicular Function

The discovery of leptin in 1994 opened an exciting field of intense research, focused on the endocrine function of the adipocyte, and conferred to adipose tissue the attribution of an endocrine organ. Leptin is a pleiotropic cytokine-like hormone that is involved in the regulation of energy homeostasis, neuroendocrine function, immunity, lipid and glucose homeostasis, and fatty acid oxidation [15, 16]. Importantly, in normal condition, circulating levels of leptin are positively correlated with adiposity [17]. On the other hand, leptin-deficient mice carrying an homozigous mutation disrupting leptin gene (*ob/ob *mice) are hyperphagic, show lower energy expenditure at rest, and are less active, and then show a severe form of obesity [18] due to the lack of leptin signalling to the brain. Of course in such condition, leptin levels are virtually absent and do not correlate with fat mass. Furthermore, *ob/ob* mice are sterile and have an abnormal spermatogenesis because of an insufficient hypothalamic-pituitary drive with consequent low circulating gonadal steroids [19, 20]. Leptin treatment normalizes body weight and restore reproduction capacity in *ob/ob *males [20]. Interestingly, it has been shown that obesity *per se* is not the cause of infertility in leptin deficiency because caloric restriction does not restore fertility in the *ob/ob* mouse. This suggests that leptin is directly related to the modifications of reproductive capacity [21]. In line with these studies, in humans, endogenous leptin absence is associated with hypogonadism and absence of pubertal development [22, 23]. Moreover, a mutation in the *db* gene that encodes leptin receptor (Ob-R) leads to the synthesis of truncated leptin receptor that lacks the intracellular domain [24]. The *db/db* mouse has an alterated reproductive function similar to those of the *ob/ob* mouse, but given that the defect is at the receptor level, leptin treatment is unable to either restore fertility or modify the appetite of these animals [25]. Hoggard et al. first identified, by *in situ* hybridization, the expression of Ob-R in the spermatic cells and Leydig cells [26]. It has been shown that leptin enters the testis by a passive, nonsaturable process [27]. A large body of evidence indicates that leptin modulates the paracrine network [28, 29] that controls gonadotropin-stimulated testicular steroidogenesis [30]. Immunohistochemical studies demonstrated that mouse testis germ cells express a functional Ob-R capable of signal transduction [31]. These data suggest that leptin can mediate proliferation and differentiation of germ cells and then might be locally involved in the pathogenesis of infertility observed in leptin-deficient mice [31]. In a recent study, a group of proapoptotic-related genes, that may play an important role in mediating the increased germ cell apoptosis and impaired sperm production, has been identified within the testes of leptin-deficient mice [32]. This study suggests a fundamental role of leptin signalling within the test is in the control of spermatogenesis.

On the other hand, excess of leptin has a negative impact on Leydig cells function [28]. In fact, treatment of cultured rat Leydig cells with leptin strongly inhibits hCG stimulated testosterone production in a dose-dependent manner. Importantly, this effect occurs at concentrations within the range of circulating levels in obese men [25]. Disruption of steroidogenic pathway occurs at the level of 17–20 lyase, as shown by the concomitant accumulation of metabolites upstream of this enzymatic step [28]. Obese patients have reduced androgen concentrations, and this reduction is related to the increase of fat mass [33] and leptin levels [34]. Moreover, the androgen response to human chorionic gonadotropin (hCG) stimulation is impaired in obese men, and leptin is the best hormonal predictor of reduction to androgen response related to obesity [35]. On the other hand, no association between leptin and dihydrotestosterone circulating levels has been observed [36].

 These observations suggest that leptin excess might have an important role in the hypogonadism, frequently observed in obese men, through a direct inhibition of Leydig cell steroidogenesis [25]. We have hypothesized that leptin acts through different sites and that there are different concentration thresholds for distinct effects of leptin on reproduction [25]; thus, a narrow range of circulating concentration of leptin are necessary in order to maintain a physiological reproductive function, and concentrations below or above these thresholds have a negative impact on hypothalamus-pituitary axis (lower threshold), or upon Leydig cell steroidogenesis (higher threshold).

## 3. Androgens, Fat Metabolism, and Adipose Biology

Testosterone is the major circulating androgen and is present in plasma as free or unbound testosterone, albumin-bound, and sex hormone-binding globulin [SHBG]-bound. In lean men, about 50% of testosterone is bound to albumin and other proteins, 44% is bound to SHBG, and 2% is unbound [37]. The biologically active component that is readily available to the tissues (bioavailable testosterone) is the proportion of unbound testosterone together with the albumin-bound fraction. A study showed that bioavailable testosterone is positively related to muscle strength and total body bone mineral density and negatively related to fat mass in healthy elderly men [38]. The fraction of testosterone bound to SHBG in serum is proportional to the SHBG levels. SHBG production in the liver is regulated by several factors and hormones, and its levels are increased by estrogen and downregulated by obesity and insulin resistance conditions [39].

Androgens influence gene transcription through the activation of the androgen receptor (AR), a ligand-activated transcription factor that binds specific DNA motifs in its target genes [40]. The extension of the polymorphic polyglutamine (CAG repeat number) of the exon 1 of the AR modulates androgen effects: androgen-induced target activities are attenuated according to the length of triplet residues [41]. Such polymorphism can influence the activity and the expression of AR and plasma androgen concentration, directly contributing to the prevalence of central adiposity [42, 43]. In particular, the CAG repeat polymorphism in the androgen receptor gene could modulate body fat mass and serum concentrations of leptin and insulin in men through a direct effect upon adipocyte sensitivity to androgens. Phenotypic effects on body fat mass could be explained by estrogen action more than androgen action, because of the increased estrogen/androgen ratio in the presence of higher CAG length; in fact, in a normal functioning adult hypothalamic—pituitary—gonadal axis, a reduced testosterone feedback, in case of a long AR CAG repeat, is compensated by increased androgen production, because of increased LH stimulation, with subsequent higher conversion to estrogens [44].

Testosterone can act directly or be converted to the more potent androgen 5-dihydrotestosterone (DHT) by 5*α*-reductase or to estrogens by aromatase (ARO) [45, 46]. ARO activity has been detected in adipose tissue [47], and several studies have demonstrated a potentially important role for this enzyme in obesity, central fat accumulation, and metabolic syndrome (MetS) [48], through estrogen receptors (ERs) and ARs, which are abundantly expressed in the adipocyte and share related functions to suppress adipose tissue accumulation and improve insulin sensitivity [49]. In a recent study, a marked decline in serum leptin levels after short-term aromatase inhibition in healthy young and elderly men has been observed [50].

A line of evidence has been reported that strongly suggests the involvement of estrogen in lipid metabolism in the adipose tissue. Fat mass is increased in male mice with homozygous inactivation of either the estrogen receptor gene or aromatase gene, and estrogen replacement is able to restore normal conditions in these models [51, 52]. At a molecular level, it has been shown that estrogens suppress fat accumulation and lipoprotein lipase (LPL, a key regulating enzyme for energy metabolism, catabolizing plasma triglycerides into free fatty acids and glycerol) mRNA expression in 3T3-L1 cells stably expressing the ER.

Obesity is associated with physiological changes which include important modifications in circulating sex steroids levels. In particular, obese men show increased plasma levels of estrogens and decreased bioavailable levels of androgens. This is due to an increase in ARO activity that mediates peripheral conversion of androgens to estrogens [53]. Circulating values of total testosterone should not be lower than 8 nmol/L (230 ng/dL). Values below this cut-off are associated with severe impairments of body composition and glucose metabolism [54–57].

Finally, adipose tissue is able to affect gonadotropin release by the pituitary, both directly, through increased secretion of cytokines, in particular TNFa [58], or indirectly, by increased conversion of circulating androgens into estrogens, which are known to decrease LH pulse [59].

## 4. Hypogonadism and Metabolic Syndrome: Clinical Evidence

Excess visceral fat and related comorbidities define a condition named metabolic syndrome, characterized by hypertension, obesity, dyslipidemia, type 2 diabetes, and insulin resistance [60, 61].

It is well established that testosterone deficiency frequently results in loss of libido and erectile dysfunction, which can be easily restored by androgen replacement therapy. Moreover, androgens directly or indirectly affect every body compartment outside reproductive organs including body composition, bone density, physical and cognitive function [62].

Patients with testosterone levels below 8 nmol/L (230 ng/dL) benefit from testosterone replacement, and testosterone administration is considered if total serum testosterone level is between 8 and 12 nmol/L, and symptoms and signs suggestive of testosterone deficiency (obesity, hypertension, dyslipidemia, insulin resistance, erectile dysfunction, decreased muscle mass and strength, decreased bone mineral density, and depressed mood) are present [63].

In recent years, several lines of evidence focused on the frequent association of hypogonadism, obesity, and MetS. Patients with MetS show significantly lower testosterone plasma levels in comparison with healthy individual [54–57, 64, 65]. Furthermore, MetS is associated with low testosterone levels independently from the criteria applied, supporting the concept that MetS can be considered as an independent risk factor for male hypogonadism [66]. In addition, prospective studies have demonstrated that low testosterone levels predict the development of diabetes and MetS [67–69]. In this context, low plasma levels of testosterone and SHBG may be early markers of MetS in nonobese men, providing a warning sign in normal weight men, considered at lower risk of developing MetS [67–69].

On the other hand, low testosterone levels could contribute to the accumulation of excess fat, establishing a vicious cycle. In fact, hypogonadism is known to induce (a) a muscle mass reduction and visceral fat mass increase, (b) insulin resistance, and (c) an increase of the activity of lipoprotein lipase (LPL), the main enzymatic regulator of triglyceride uptake in the fat cell, preferentially in abdominal fat [62].

Interesting studies evaluated body composition changes in men undergoing androgen deprivation therapy for nonmetastatic prostate cancer. 12 and 48 weeks of androgen deprivation determined a significant increase of BMI and fat mass [70] and an increased incidence of diabetes and cardiovascular disease [71].

Rapid weight loss with successful weight maintenance in obese men with MetS induced a sustained increase in free testosterone levels [72]. Also, testosterone treatment of obese, insulin-resistant, nondiabetic and diabetic men has been shown to reduce fat mass, increase lean body mass, decrease waist circumference, which represents a valid parameter of the degree of visceral obesity, finally improving HOMA index and glycemic control [58, 66].

The frequent association of hypogonadism and obesity has led a group of experts to formulate the following recommendation: patients with clinical conditions associated with insulin resistance (obesity, type 2 diabetes, MetS) should be screened for testosterone deficiency, given that such conditions often coexist [63].

## 5. Effects of Low Testosterone Levels on Body Fat Mass

**Figure 1:**
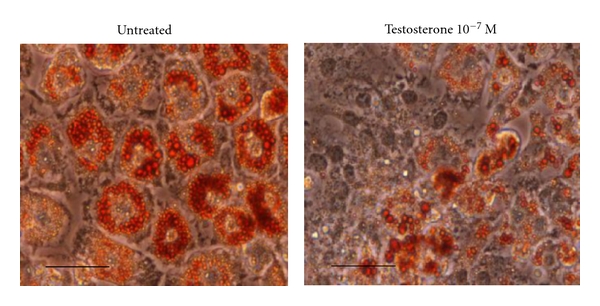
Effect of testosterone on 3T3-L1 adipose differentiation. Red oil staining of mature 3T3-L1 adipocytes (scale bar, 70 *μ*m) (Caprio M. et al., unpublished), classically differentiated in the absence (left) or in the presence (right) of 10^−7^ M testosterone. Testosterone treatment determines a marked reduction in size and number of lipid droplets, in accordance with previous reports [73].

As shown in the previous paragraph, several lines of evidence strongly suggest that androgens influence body fat distribution and accumulation (see Figure 1). Men affected by androgen resistance due to gene inactivation of the AR show high visceral fat [43]. Male mice lacking AR develop obesity with increased lipogenesis in WAT and liver [74, 75]. In males, the antiobesity action of testosterone might be indirectly mediated via AR signalling in skeletal muscle. In fact, testosterone promotes the commitment of pluripotent cells of mesenchymal origin into myogenic lineage *in vitro*, by inhibiting adipogenic differentiation. These effects are mediated through an AR-dependent mechanism [76]. The same authors a few years later demonstrated that in 3T3-L1 preadipocytes, AR modulates adipogenic differentiation by directly activating downstream Wnt effector molecules, including *β*-catenin, T-cell factor (TCF), and lymphoid-enhancer factor (LEF) [73] (see Figure 1).

Recently, we have demonstrated that Drospirenone (DRSP), a progestogen with a modest antiandrogenic activity, widely used for contraception [77–79], strongly inhibits adipose differentiation both in murine (3T3-L1) as well as in human preadipocytes *ex vivo*. It is important to remark that DRSP is a powerful antagonist of the mineralocorticoid receptor (MR), which is a pivotal factor for the induction of adipogenesis. We have shown that the antiadipogenic effect of DRSP relies on specific antagonism on the MR [80]. Surprisingly, DRSP antiadipogenic effect is blunted in presence of testosterone, whereas we could have predicted a synergic effect on the inhibition of adipogenesis. In order to explain these data, we hypothesize that chronic treatment of preadipocytes *in vitro* with testosterone could upregulate AR, as already shown in different cellular models [81, 82]. As a consequence, increased levels of AR may bind DRSP as an AR antagonist, and the overall availability of DRSP as an anti-MR could result reduced.

AR activation in skeletal muscle might indirectly decrease WAT mass through increased muscle oxidative metabolism or through the release of an unknown circulating factor [49]. Indeed, in muscle cells of transgenic male rats overexpressing AR, increased lean mass with hypertrophy of type IIb fibers, increased oxidative metabolism, and decreased adipocyte size and WAT mass are observed [83]. However, adipose-specific AR knockout mice are not obese and show increased WAT production of leptin without leptin resistance [84]. Authors conclude that in adipocytes AR plays an inhibitory role in leptin production [84], but lack of androgens signalling in the adipocyte is not sufficient to promote obesity. Probably, the adipocyte is not the only player in the complex regulation of fat metabolism, and other cell types in its context could represent important targets of androgens. Tissue-specific inactivation of AR is deemed necessary to clarify these aspects.

## 6. Future Perspectives and Conclusions

**Figure 2:**
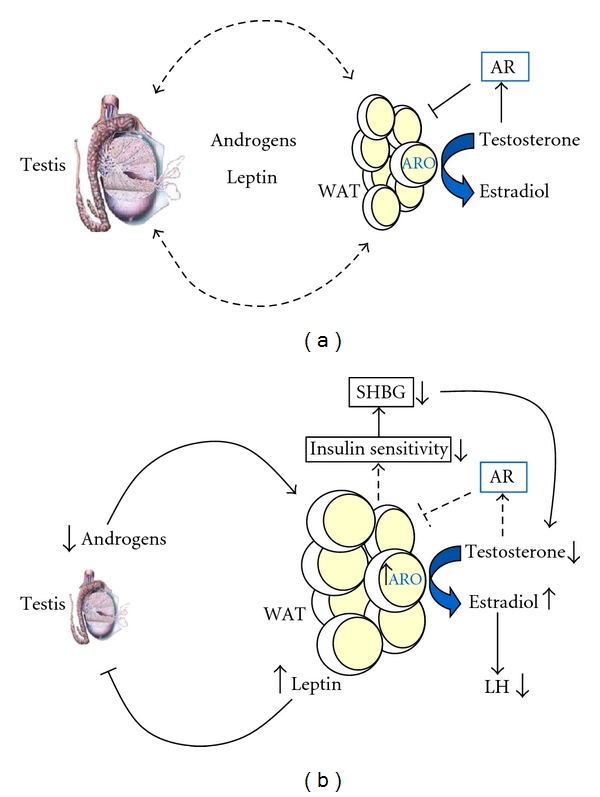
Reciprocal interplay between white adipose tissue (WAT) and testis. (a) In normal conditions, circulating androgens control adipocyte size and adipose mass. On the other hand, plasmatic leptin, mainly produced by adipose tissue, regulate testicular steroidogenesis. Dashed bars indicate the reciprocal interplay between testis (through androgens) and adipose tissue (mainly through leptin). (b) Androgen deficiency induces expansion of fat mass and subsequent dysregulation of several functions controlled by adipose tissue such as insulin sensitivity, blood pressure, vascular reactivity, and immunity. The state of insulin resistance determined by obesity leads to reduced production of SHBG. The consequent reduction in testosterone triggers expansion of adipose mass with subsequent increase in aromatase (ARO) activity, which in turn mediates peripheral conversion of testosterone to estradiol. Increased estrogen levels induce a reduction of LH pulse which contributes to the reduction in androgen production. On the other hand, excess of circulating leptin, due to increased adipose mass, disrupts testicular steroidogenesis, with consequent suppression of androgen production. The vicious cycle is triggered. AR: androgen receptor.

Experimental and clinical studies indicate that adipose tissue and gonads communicate and influence each other, either directly or indirectly, through several circulating factors (see Figures 2(a) and 2(b)). Obesity is often associated with low plasma-testosterone levels and reproductive dysfunction, given that low testosterone may not be necessarily observed in human obesity, nor be the only cause of visceral obesity. This can be due to excessive circulating levels of leptin, which have been shown to disrupt the steroidogenic function of Leydig cell, with a subsequent reduction in hCG-driven testosterone production. Other adipokines, together with leptin, could play a direct or indirect role in the alteration of Leydig cell function in obesity.

On the other hand, circulating levels of sex hormones control fat mass distribution and expansion, mainly through activation of estrogen and androgen receptors in adipose tissue. Of interest, a recent work highlighted the profound impact of testosterone on cardiovascular function improving functional capacity, heart rate, muscle strength, and glucose metabolism in elderly patients with coronary heart failure [85]. We hypothesize that the cardiovascular effects of testosterone may be also mediated by adipose tissue, which embeds the heart and the most important vessels (coronaries, carotids, aorta, etc.) and is an active site of conversion of androgens into estrogens, through aromatase activity.

In conclusion, adequate levels and balance of circulating sex hormones are necessary to maintain a correct distribution and size of adipose tissue, which in turn is fundamental to keep a normal reproductive and sexual function. For this reason, screening of obese patients for hypogonadism is deemed necessary in order to better understand the pathophysiology of coexistent metabolic alteration, in order to target it with a replacement therapy. The delicate issue of whether testosterone decline, observed with aging, causes adipose tissue accumulation, or whether weight gain primarily disrupts testicular steroidogenesis, is still unclear and needs further studies.
